# Health Professions' Educators' Preparedness for Inclusive Education: The HEPIE Study

**DOI:** 10.1111/tct.70364

**Published:** 2026-02-17

**Authors:** Gisselle Gallego, Chris Forlin, Lise Mogensen, Laura Gray, Claudia Ng, Kim Bulkeley, Aishah Moore, Dinesh Palipana

**Affiliations:** ^1^ School of Medicine The University of Notre Dame Australia Sydney New South Wales Australia; ^2^ Centre for Disability Studies, an affiliate of the University of Sydney Camperdown New South Wales Australia; ^3^ School of Education The University of Notre Dame Australia Fremantle Western Australia Australia; ^4^ School of Medicine Western Sydney University Campbelltown New South Wales Australia; ^5^ The Damion Drapac Centre for Equity in Health Professions Education, Faculty of Health Deakin University Victoria Australia; ^6^ The Centre for Research in Assessment and Digital Learning (CRADLE) Deakin University Victoria Australia; ^7^ Sydney School of Health Sciences The University of Sydney Camperdown New South Wales Australia; ^8^ Faculty of Health Sciences & Medicine Bond University Robina Queensland Australia; ^9^ Emergency Department Gold Coast University Hospital Gold Coast Queensland Australia

**Keywords:** Australia, disability, education, health professions, inclusion

## Abstract

**Background:**

To achieve a healthcare workforce that reflects the diversity of the communities it serves, we need to ensure that students with disability have access to safe, effective educational experiences in health professions education. This study aimed to explore health professions' educators (HPEs) views on inclusive education, acceptance of students with different needs and comfort levels when engaging with students with disability.

**Methods:**

An online survey of Australian HPEs was conducted between June and November 2024. Two scales were used to measure preparedness and concerns regarding teaching students with disability. Analyses of variance were performed to compare the mean difference in both scales. The relationship was assessed using a linear regression model.

**Results:**

A total of 148 HPEs completed the survey; of these, 72% were females, 64% were employed in a part‐time capacity and on average had been teaching for 12.6 years. They perceived that they had some confidence, knowledge and resources for teaching students with disability, but reported that they had received limited training. HPEs were most concerned about students not being accepted by healthcare or clinical educators and the difficulty of providing appropriate support.

**Discussion:**

HPEs had limited training and were concerned that students would not be accepted by healthcare or clinical educators. These findings suggest that reshaping the culture and practices of HPE with regards to disability inclusion may be required to provide HPEs with the skills they need to support students, to dispel misconceptions and to provide students with disability learning environments where they can safely and equitably thrive.

## 1 Introduction

Creating a healthcare workforce that reflects the communities it serves has long been understood as key to the provision of high quality, responsive and equitable healthcare [1]. Diversification of the healthcare workforce to improve disability inclusion is, therefore, essential [2, 3]. Approximately 16% of the world's population has a disability [4], while in 2022, more than 21% of the Australian population (one in five) reported having a disability [5]. Health care providers with disability are, however, underrepresented in both the Australian and the international healthcare sector [6, 7]. The underrepresentation of people with disability^1^ in health professions demonstrates the lack of workforce diversity and has the potential to increase unconscious bias in health service delivery [6]. Despite legislative support, people with disability continue to face barriers to education and employment, including segregation, inaccessible environments, lack of accommodations, inadequate support, social exclusion and ongoing ableism [7, 8].

Effective inclusion of students with disability in health professions education requires identification of potential barriers to participation in both preclinical and clinical environments and an understanding of how these can be addressed in feasible and practical ways making health education accessible and inclusive. Prior to its application in higher education, inclusive education was originally developed for children and younger students [9]. One of the challenges, however, has been to implement the principles of inclusive education in higher education [9] and particularly in health professions education. Inclusive health education has been described as ‘…active engagement of all members of the learning community’ [10] (p 1). Davis [11] states that in this context it ‘…means they are fully included in programs, with their contributions valued and disability being viewed as one form of diversity’ (p 1).

International research studies regarding health professions education have shown that students with disability can encounter stigma, a lack of institutional openness to admission, a lack of clear policy and procedure, a lack of access to appropriate support and inappropriate or inadequate accommodations [12, 13]. Furthermore, studies have found that students with disability who enter and successfully complete healthcare programs face discrimination and ableism when employed and may be viewed as a safety risk [11, 14, 15]. Research has also shown that one of the main barriers faced by students with disabilities is educators [16, 17, 18]. However, there has been limited research exploring whether health professional educators (HPEs) are prepared for inclusive education, engaging with students with disability, or know how to implement inclusive practices in health professions education. A qualitative study in Norway that included healthcare educators found that there was a lack of knowledge of how to accommodate students [19]. A review by Lawlis et al. [20] found that negative attitudes of university staff and clinical educators were associated with students experiencing discrimination in work integrated learning (WIL) experiences such as clinical placements.

As the number of students with disability enrolling in postsecondary institutions and healthcare programs is on the rise both internationally and in Australia [21]. Hence, it is crucial to assess whether HPEs are adequately prepared to identify their training needs to effectively support these students. The aim of this study was to address this knowledge and practice gap by exploring HPEs' views on inclusive education, acceptance of students with different needs and comfort levels when engaging with students with disability.

## 2 Materials and Methods

### 2.1 Study Design and Participants

This study design was cross‐sectional using an online survey of HPEs based in Australia and teaching between June and November 2024. Because there is no universally agreed definition for HPE, for the purpose of the survey, HPEs were defined as a professional involved in the development, education and training of health professionals, including clinical education. Invitations were circulated via the authors' faculties and schools, the Australian and New Zealand Association for Health Professional Educators (ANZAHPE) e‐newsletter, postgraduate health education courses and social media.

### 2.2 Sample Size

This was a descriptive, exploratory study; a pragmatic sample of respondents was used to test the validity of the survey instrument as well as the reliability of the modified Sentiments, Attitudes and Concerns about Inclusive Education Revised (SACIE‐R) scale. Utilising a nonprobability convenience sample, a total of 148 responses were deemed suitable for analysis.

### 2.3 Questionnaire

The online survey included a multiple‐choice and 5‐point Likert scale questionnaire consisting of four sections. Section one asked HPEs demographic questions including gender, age, whether they were from a culturally and linguistically diverse (CALD) background and whether they identified as having a disability themselves. It also asked their employment status, whether they had completed a qualification in education, major field of teaching and the duration of their teaching experience. Respondents were also asked to evaluate the extent to which they had interacted with people with disability and their level of experience teaching students with disability.

The second section was the adapted version of two scales. The first scale—Preparedness for Teaching Students with Disability—consisted of a 5‐point Likert scale with seven items exploring respondents' perceived preparation and capabilities for teaching students with disabilities. Items were derived from the literature regarding reported aspects of best practice training for inclusion and adapted to health education [22, 23, 24]. The third section included an adapted version of the SACIE‐R [25]. This scale was developed to measure preservice teachers' perceptions about inclusion. In the preservice teacher context, ‘inclusive education’ is used to describe the inclusion of children and young people with special educational needs [26]. Hence, the scale was adapted to use with HPEs. This is the first study to use the scale in the HPE context. For the purposes of this study, the authors, including the developer of the original scale (CF), adapted the five items in the SACIE‐R scale relating to concerns about inclusive education by modifying the language slightly to reflect the health professions education setting (see Supplementary material). The two Likert scales required responses from strongly disagree (assigned a value of 1), somewhat disagree (2), neither agree nor disagree (3), somewhat agree (4), to strongly agree (5). A higher mean score indicated a greater preparedness to teach (Scale 1) or an increased level of concern regarding teaching students with disabilities (Scale 2).

The fourth section had open‐ended questions about HPEs' perceived education gaps and limitations at an institutional level, as well as educational needs and support. The questionnaire was pilot‐tested for clarity of wording, content, comprehensibility, feasibility and face validity with HPEs from different disciplines in the researchers' network.

### 2.4 Data Collection

The online survey was distributed using the Qualtrics platform. Completion time was approximately 15–20 min, and the survey was open for a 6‐month period in 2024. Participation was voluntary and anonymous, with participants providing consent prior to conducting the survey. Participants were unable to withdraw their data after submitting as it was recorded anonymously. Participants were not remunerated but were provided with a chance to win one of four gift vouchers.

### 2.5 Statistical Analysis

Data were cleaned and checked to remove any incomplete surveys. Where incorrect responses were included, these were marked as missing. Descriptive statistics were used to summarise the data by reporting the frequencies and proportions of the demographic and teaching factors. We estimated the overall (total) mean across all questions for each participant in both preparedness to teach (seven items) and concerns with teaching scales (five items). We tested the normality distribution of both total mean scales by Shapiro–Wilk test (*p* < 0.05). We reported the mean and SD of both total scales. Analyses of variance (ANOVA; *p* < 0.05) were performed to compare the mean difference in both scales between each demographic and teaching factor groups. We assessed the relationship of the demographic and teaching factors with both total mean scales (continuous) using linear regression model to estimate the unstandardised and standardised coefficients and their 95% confidence intervals (CI) and *P*‐value. The categorical variables were recoded into dummy coding (binary 0/1) and included in the model. Missing data were excluded from the analysis for each variable. Responses were analysed using the Statistical Package for the Social Sciences (SPSS) for Windows Version 29 (SPSS Inc., Chicago, IL, USA).

## 3 Results

### 3.1 Respondent Variables and Overall Outcomes for Preparedness to Teach and Concerns With Teaching

A total of 148 complete responses were recorded in the survey. The response rate could not be estimated as the survey distribution was open. Of the cohort, 40 respondents (27%) identified as having a disability, 107 (72%) were female and 54 (37%) were from a CALD background. Their ages ranged from 25 years to greater than 50 years (44% of participants ranged between 35 and 45 years of age). Table 1 indicates the responses for demographic information and Table 2 reports the teaching variables. The mean responses to the two scales were calculated for all levels of the demographic (Table 1) and teaching variables (Table 2).

**TABLE 1:** Frequencies of demographic variables for preparedness to teach (*N* = 148) and concerns with teaching (*N* = 145).

	Preparedness to teach *N* = 148			Concerns with teaching *N* = 145		
Characteristic	*N* (%)	Mean (SD)*	ANOVA *p* **	*N* (%)	Mean (SD)*	ANOVA *p* **
Total	148	3.30 (0.69)		145	3.16 (0.71)	
Identified as having a disability						
Yes	40 (27)	3.18 (0.64)	0.16	40 (28)	3.13 (0.58)	0.74
No	106 (73)	3.35 (0.70)		103 (72)	3.17 (0.77)	
Gender						
Male	40 (27)	3.12 (0.74)	0.08	38 (26)	3.16 (0.46)	0.94
Female	107 (72)	3.37 (0.66)		107 (74)	3.15 (0.78)	
Prefer not to answer	1 (1)	—		0		
Age groups (years)						
25–29	12 (8)	3.40 (0.52)		11 (8)	2.82 (0.69)	
30–34	24 (16)	3.39 (0.57)		23 (16)	3.17 (0.72)	
35–39	34 (23)	3.21 (0.65)	0.94	33 (23)	3.19 (0.73)	0.55
40–44	31 (21)	3.27 (0.72)		31 (21)	3.25 (0.64)	
45–49	11 (8)	3.30 (0.82)		11 (8)	3.33 (0.73)	
> 50	36 (24)	3.29 (0.80)		36 (24)	3.08 (0.76)	
CALD						
Yes	54 (37)	3.22 (0.73)	0.31	52 (36)	3.15 (0.67)	0.94
No	94 (63)	3.34 (0.67)		93 (64)	3.16 (0.74)	

*Note:* Means of Likert scale responses ranging from strongly disagree (1), somewhat disagree (2), neither agree nor disagree (3), somewhat agree (4) to strongly agree (5). CALD, culturally and linguistically diverse.

* Total mean scores for Likert scale responses.

** Significant at *p* < 0.05.

**TABLE 2:** Frequencies of teaching variables for preparedness to teach (*N* = 148) and concerns with teaching (*N* = 145).

	Preparedness to teach *N* = 148			Concerns with teaching *N* = 145		
Characteristic	*N* (%)	Mean (SD)*	ANOVA *p*‐value**	*N* (%)	Mean (SD)*	ANOVA *p*‐value**
Total	148	3.30 (0.69)		145	3.16 (0.71)	
Employment						
Full‐time	54 (37)	3.59 (0.75)	< 0.001	54 (37)	3.10 (0.81)	0.47
Part‐time	94 (63)	3.13 (0.59)		91 (63)	3.19 (0.65)	
Education qualification						
Yes	92 (62)	3.36 (0.65)	0.14	92 (63)	3.16 (0.72)	0.91
No	56 (38)	3.19 (0.74)		53 (37)	3.15 (0.71)	
Considerable interactions with people with disability						
Yes	113 (76)	3.38 (0.69)	0.008	113 (78)	3.13 (0.67)	0.50
No	35 (24)	3.03 (0.62)		32 (22)	3.23 (0.85)	
Level of experience teaching learners with disability						
Nil	16 (11)	2.87 (0.61)		13 (9)	3.17 (0.65)	
Some	123 (83)	3.27 (0.63)	< 0.001	123 (85)	3.18 (0.71)	0.30
High	9 (6)	4.37 (0.51)		9 (6)	2.80 (0.81)	

*Note:* Means of Likert scale responses ranging from strongly disagree (1), somewhat disagree (2), neither agree nor disagree (3), somewhat agree (4), to strongly agree (5).

* Total mean scores for Likert scale responses.

** Significant at *p* < 0.05.

More HPEs were employed in a part‐time capacity (94, 63.5%), compared to full‐time (54, 36.5%). More than half of the cohort (92, 62.2%) had completed a qualification in education, and the majority had at least some experience with teaching, with many stating that they had considerable interactions with students with disability. The area of health education in which the respondents were teaching varied considerably. The majority were teaching in medicine (29, 19.6%) or nursing and midwifery (28, 18.9%). On average, the respondents had been teaching for 12.62 years (SD = 8.97), with a range from 1 to 46 years.

### 3.2 Responses to Individual Questions in Each Scale

All participants (*N* = 148) responded to all individual items in the Preparedness for Teaching Students with Disability scale. The overall mean scale was normally distributed (Shapiro–Wilk test *p*‐value = 0.40) and the overall mean was 3.30, SD 0.69.

Table 3 shows the responses to each question assessing this scale, ordered from highest to lowest mean response. Respondents indicated overall positive experiences working with students with disability (M = 3.67, SD 1.02) and reported that they often seek additional guidance in how they can best assist them (M = 3.50, SD 1.18). They perceived that they had some confidence, knowledge and resources for teaching students with disability, but reported that they had received limited training (M = 2.63, SD 1.29). ANOVA showed that there were no statistically significant differences in the overall mean preparedness to teach scale between groups based on the demographic factors (Table 1). For teaching factors, our findings showed that there was a statistically significant higher mean scale among participants with high level of experience (M = 4.37, SD 0.51) than lower levels (some M = 3.27, SD 0.63; nil M = 2.87, SD 0.61; *p* < 0.001; Table 2). Participants working full‐time had a statistically significant higher mean scale compared to those working part‐time (*p* < 0.001; Table 2).

**TABLE 3:** Distribution of items in the preparedness for teaching learners with disability scale, rank ordered from highest to lowest levels of perceived preparedness according to the total item mean (*N* = 148).

	1		2		3		4		5		Mean	SD
Statement	*N*	%	*N*	%	*N*	%	*N*	%	*N*	%		
I have had positive experiences	6	4.1	11	7.4	40	27.0	60	40.5	31	20.9	3.67	1.02
I often seek additional guidance	13	8.8	17	11.5	29	19.6	61	41.2	28	18.9	3.50	1.18
I understand the legal requirements	13	8.8	24	16.2	19	12.8	61	41.2	31	20.9	3.49	1.24
I am prepared to teach	5	3.4	36	24.3	24	16.2	60	40.5	23	15.5	3.41	1.12
I have confidence in teaching	14	9.5	31	20.9	31	20.9	52	35.1	20	13.5	3.22	1.20
I have knowledge and resources	10	6.8	42	28.4	29	19.6	49	33.1	18	12.2	3.16	1.17
I have had training	31	20.9	52	35.1	21	14.2	29	19.6	15	10.1	2.63	1.29

*Note:* Means of Likert scale responses range from strongly disagree (1), somewhat disagree (2), neither agree nor disagree (3), somewhat agree (4), to strongly agree (5).

One hundred and forty‐five participants responded to all individual items in the ‘Concerns about Teaching Students with Disability scale’ (five items). The overall mean scale was normally distributed (Shapiro–Wilk test *p*‐value = 0.40) and the overall mean was 3.16 (SD 0.71). Table 4 shows the responses to each question in the scale, ordered from highest to lowest mean response. Participants were less concerned about there being an increase in their workload (M = 3.17, SD = 1.24), or that they would be more stressed if they had students with disability in their classes (M = 2.71, SD 1.25). ANOVA results showed that there were no statistically significant differences in the overall mean concerns for teaching scale between groups of the demographic and teaching factors (Table 1 and Table 2). There was a lower overall mean scale for those participants with high level of experience compared to lower level of experience. However, this difference was not statistically significant (*p* = 0.30; Table 2).

**TABLE 4:** Distribution of items in the ‘Concerns about Teaching Learners with Disability’ scale rank ordered from highest to lowest levels of concern according to the total item mean (*N* = 145).

Statement	1		2		3		4		5		Mean	SD
I am concerned that:	*N*	%	*N*	%	*N*	%	*N*	%	*N*	%		
Learners will not be accepted	13	8.97	22	15.2	24	16.6	60	41.4	26	17.9	3.44	1.21
It will be difficult to give support	6	4.14	37	25.5	30	20.7	59	40.7	13	9.0	3.25	1.06
I do not have the knowledge and skills	15	10.3	29	20.0	29	20.0	54	37.2	18	12.4	3.21	1.20
My workload will increase	14	9.66	36	24.8	29	20.0	44	30.3	22	15.2	3.17	1.24
I will be more stressed	27	18.6	45	31.0	30	20.7	29	20.0	14	9.7	2.71	1.25

*Note:* Means of Likert scale responses range from strongly disagree (1), somewhat disagree (2), neither agree nor disagree (3), somewhat agree (4), to strongly agree (5).

The Preparedness for Teaching Students with Disability scale had an overall mean of 3.30, SD 0.69, with a reliability index Cronbach alpha coefficient of 0.681. The Concerns about Teaching Students with Disability scale (*n* = 5) had an overall mean of 2.98, SD 0.66, with a reliability index of 0.549.

### 3.3 Assessing the Relationship Between Preparedness for Teaching Students With Disability Scale and Demographic and Teaching Factors

The demographic and teaching factors were incorporated into a linear regression model to explore their relationship with the Preparedness for Teaching scale while potentially controlling for other factors (Table 5). The model was moderately fitted and provided an explanation of the relationship (*R* = 0.56, *R* ^2^ = 0.32). The model demonstrated that participants with a high level of experience had significantly higher overall mean scale of preparedness to teach students with disability than those with lower levels of experience (*B* coefficient 1.026 [95% CI 0.596 to 1.456], *p* < 0.001). Participants working part‐time had a significant 0.313 lower overall mean scale than participants working full‐time (B coefficient −0.313 [−0.543 to −0.083], *p* = 0.008). Also, those who did not complete their education qualification were reported to have a significantly lower overall mean scale than those who completed their education qualification (B coefficient −0.246 [−0.483 to −0.008], *p* = 0.043). Younger age participants (25–29 years) had a higher overall mean scale compared to older participants; however, this was not a statistically significant association (B coefficient 0.226 [−0.191 to 0.642], *p* = 0.286; Table 5).

**TABLE 5:** Assessment of the relationship of the demographic and teaching factors with ‘Preparedness to Teach’ scale using linear regression model (*N* = 148).

Variable	Unit	B^a^	SE	Beta^b^	*t*	*p*	95% CI for B	
							Lower bound	Upper bound
Constant		3.744	0.144		25.964	< 0.001	3.458 —	4.029
Having a disability	Dummy (0 = no, 1 = yes)	−0.037	0.171	−0.024	−0.218	0.828	−0.375	0.300
Gender	Dummy (0 = female, 1 = male)	−0.116	0.116	−0.076	−0.998	0.320	−0.347	0.114
Age groups (years)								
25–29 years	Dummy (0 = others, 1 = 25–29)	0.226	0.211	0.090	1.072	0.286	−0.191	0.642
30–34 years	Dummy (0 = others, 1 = 30–34)	0.066	0.165	0.036	0.401	0.689	−0.260	0.392
35–39 years	Reference							
40–44 years	Dummy (0 = others, 1 = 40–44)	−0.106	0.154	−0.063	−0.692	0.490	−0.410	0.198
45–49 years	Dummy (0 = others, 1 = 45–49)	−0.163	0.213	−0.063	−0.763	0.447	−0.584	0.259
≥ 50 years	Dummy (0 = others, 1 = ≥ 50)	−0.064	0.155	−0.040	−0.415	0.679	−0.370	0.242
CALD	Dummy (0 = no, 1 = yes)	−0.210	0.138	−0.148	−1.529	0.129	−0.483	0.062
Employment	Dummy (0 = full‐time, 1 = part‐time	−0.313	0.116	−0.220	−2.689	0.008	−0.543	−0.083
Education qualification	Dummy (0 = yes, 1 = no)	−0.246	0.120	−0.173	−2.048	0.043	−0.483	−0.008
Considerable interactions with people with disability	Dummy (0 = yes, 1 = no)	−0.235	0.136	−0.146	−1.731	0.086	−0.504	0.034
Level of experience teaching learners with disability								
Nil	Dummy (0 = others, 1 = nil)	−0.146	0.192	−0.066	−0.759	0.449	−0.526	0.234
Some	Reference							
High	Dummy (0 = others, 1 = high)	1.026	0.217	0.360	4.720	< 0.001	0.596	1.456

*Note:* Level of significance (*p*‐value) at *p* < 0.05. Model: *F* = 4.72; df = 13; *p* < 0.001; *R* = 0.56; *R* ^2^ = 0.32.

Abbreviations: CI = confidence interval; SE = standard error.

^a^ Unstandardised coefficients.

^b^ Standardised coefficients.

Many of the HPEs had a qualification in education and these considered themselves better prepared to teach students with disability. Likewise, those who indicated having had considerable interactions with students with disability also considered themselves more prepared to teach them, although neither of these were statistically significantly different. Gender differences indicated that females considered themselves better equipped than males for teaching. Of particular interest is that the HPEs who identified themselves as having a disability felt less prepared to teach than those without a disability.

### 3.4 Assessing the Relationship Between Concerns About Teaching Students With Disability Scale and Demographic and Teaching Factors

We incorporated the demographic and teaching factors in a linear regression model to explore their relationship with the Concerns about Teaching scale while potentially controlling for other factors (Table 6). The results showed that the model was poorly fitted data and was not a strong predictor of the relationship (*R* = 0.25, *R* ^2^ = 0.10). The model demonstrated that participants with a high level of experience had 0.5 lower overall mean scale of concern with teaching than those with lower levels of experience (B coefficient −0.477 [−1.004 to 0.050]; *p* = 0.076). Younger age participants (25–29 years) had lower concern about teaching students with disability than older participants (B coefficient −0.466 [−0.998 to 0.066]; *p* = 0.085; Table 6).

**TABLE 6:** Assessment of the relationship of the demographic and teaching factors with ‘Concerns to Teach’ scale using linear regression model (*N* = 145).

Variable	Unit	B^a^	SE	Beta^b^	*t*	*p*	95% CI for B	
							Lower bound	Upper bound
Constant		3.162	0.178		17.787	< 0.001	2.810	3.514
Having a disability	Dummy (0 = no, 1 = yes)	−0.234	0.211	−0.147	−1.110	0.269	−0.651	0.183
Gender	Dummy (0 = female, 1 = male)	0.018	0.145	0.011	0.126	0.900	−0.268	0.305
Age groups (years)								
25–29 years	Dummy (0 = others, 1 = 25–29)	−0.466	0.269	−0.173	−1.733	0.085	−0.998	0.066
30–34 years	Dummy (0 = others, 1 = 30–34)	0.015	0.206	0.008	0.072	0.943	−0.392	0.421
35–39 years	Reference							
40–44 years	Dummy (0 = others, 1 = 40–44)	0.071	0.189	0.041	0.373	0.710	−0.304	0.445
45–49 years	Dummy (0 = others, 1 = 45–49)	0.121	0.261	0.045	0.464	0.644	−0.396	0.639
≥ 50 years	Dummy (0 = others, 1 = > = 50)	−0.150	0.191	−0.091	−0.789	0.432	−0.527	0.227
CALD	Dummy (0 = no, 1 = yes)	0.115	0.174	0.077	0.659	0.511	−0.230	0.459
Employment	Dummy (0 = full‐time, 1 = part‐time	0.102	0.143	0.069	0.714	0.477	−0.180	0.384
Education qualification	Dummy (0 = yes, 1 = no)	0.077	0.148	0.051	0.516	0.607	−0.217	0.370
Considerable interactions with people with disability	Dummy (0 = yes, 1 = no)	0.046	0.167	0.027	0.274	0.785	−0.286	0.377
Level of experience teaching learners with disability								
Nil	Dummy (0 = others, 1 = nil)	−0.118	0.243	−0.047	−0.484	0.629	−0.599	0.363
Some	Reference							
High	Dummy (0 = others, 1 = high)	−0.477	0.267	−0.162	−1.789	0.076	−1.004	0.050

*Note:* Level of significance (*p*‐value) at *p* < 0.05. Model: *F* = 0.68; df = 13; *p* = 0.78; *R* = 0.25; *R* ^2^ = 0.10.

Abbreviations: CI = confidence interval; SE = standard error.

^a^ Unstandardised coefficients.

^b^ Standardised coefficients.

## 4 Discussion

This study explored HPEs' views on inclusive education, acceptance of students with different needs, and comfort levels when engaging in teaching students with disability.

### 4.1 Preparedness for Teaching Students With Disability

The results show that HPEs have had positive experiences with students with disability and often seek guidance to assist them. They also understand the legal requirements to make accommodations but reported having had limited training on educating students with disability. Consistent with our findings, research has shown that in higher education, university staff report a lack of specific disability training and information [17, 27]. A recent scoping review to identify strategies that may improve health professional programs found that culture change for educators and staff is needed [28].

As suggested by Loutzenheiser and Erevelles [29], disability has historically been an outlier in education, often considered a social category where disability is ‘included in’ instead of something educators ‘engage’ with. Universitas 21 Network (U21; a global network of universities with a strong commitment to equity, diversity and inclusion) member universities found that staff‐focused disability awareness training varied significantly within these institutions, with many not requiring any training for staff [30]. The report recommended an expansion of mandatory training for staff and students, with the expectation that this provides a baseline level of understanding and illustrates the commitment to inclusion of the organisation. Trials of higher education faculty training programs have demonstrated positive impacts on educator knowledge and motivation regarding supporting students with disability [31, 32]. Importantly, reviews of disability inclusion in health professions education also point to the role of faculty training on shifting the prevailing cultural attitudes towards disability in students and the healthcare workforce [28].

### 4.2 Concerns About Teaching Students With Disability

The most significant concern for HPEs in this study was the perception that students with disability would not be accepted by healthcare or clinical educators. Research in health education has shown that ‘competencies’ for both clinical practice and academic context are one of the most common forms of discrimination. As described by Jarus et al. [33], this occurs because competency expectations are embedded in the medical model of disability that sees disability as an individual issue. This means that ‘safe practice’ is associated with an ‘able body’ and considers that people with disability are likely to be seen as vulnerable, risky and unsafe. Their ability to provide safe care is questioned; this is despite no research evidence to support this idea. Research has shown that students with disability in health are faced not only with systemic barriers that occurred by disabling institutions and systems, but also by perceptions about their competence [34]. In health education and health professions, the notions of competence and capability have been widely adopted. Described as the ‘capability imperative’ HPEs often refer to the ability of students to practice in the ‘real world’ where it is perceived that accommodations and supports are less likely to occur [35]. According to Ashcroft and Lutfiyya [36], nursing educators described that they expect to produce ‘competent graduates’. Research with health professionals in Canada found differences in perspectives between academic coordinators and field educators [34]. Compared to field educators, academic coordinators were somewhat more negative about students who required accommodations. Furthermore, academic coordinators thought there was a potential burden associated with students who need accommodations that could harm placement relationships with fieldwork sites [34].

HPEs in our study were less concerned about there being an increase in their workload, or that they would be more stressed if they had students with disability in their classes. Importantly, levels of prior experience and full‐time employment led to increased preparedness to teach, which might suggest that the exposure and practical experience of working with students with disability at least partially offset the lack of specific training in this area. However, this raises the challenge of how early‐career HPEs or those who have not had significant opportunities to work with students with disability might be enabled to establish the knowledge, skills and confidence to provide appropriate learning environments and support for students with disability. As participants in this survey have identified, training and professional development might play a key role. These findings suggest a generally positive attitude of HPEs towards supporting students with disability, which might contrast with studies in other educator populations where the barriers identified include lack of time, buy‐in, unwillingness and fear of change [37].

### 4.3 Future Directions

When interpreting these results, it is critical to consider that even though statistically significant differences were achieved between HPEs according to teaching experience and whether they were working full or part‐time, only noticeably minimal differences between the means for each item in the scales were to be found. Further consideration should also be given to not just the overall results but the meaning for individuals who do not report the average or mean responses for preparedness and concerns. Although the mean response, which is used here to identify potential issues, is useful in providing a generic overview, this does not consider the obvious disparity between responses. Future attention will need to consider potential variables that might provide a clearer understanding of these differences between HPEs' responses so that more directed and focused support may be provided to ensure improved preparation for teaching students with disability for all HPEs. This will require targeted, carefully designed and well‐supported professional development and training, not tokenistic additions on top of a full teaching portfolio. As Dhillon et al. [28] comment in their recent review, educators need to both be provided with an understanding of disability from a critical perspective, and opportunities to ‘reflect on personal biases and current practices in comparison to their new learning’ to enable action towards culture change. There is evidence to support the impact of this holistic approach to educator professional development, showing that following training educators are better equipped to take practical steps, but also have a different ‘way of thinking about what disability means’ [32].

Identifying and understanding current structural and attitudinal barriers to inclusive health education is a vital stepping stone to developing, advancing and disseminating leading practices to facilitate the inclusion of people with disability in health professions education.

Creating a culture and climate towards disability inclusion in health professional education will broaden the diversity of the health professions' workforce and by extension, improve healthcare outcomes for people with disability.

## 5 Limitations

Studies such as these have several limitations. First, the wording of questions and the way they are presented influences participant responses. Second, because of the small number of HPEs that participated in this survey, it is not possible to generalise the study findings to all HPEs in Australia. It is also likely to be over‐representative of those with an interest in the research topic. However, the gender difference was seen as being representative of HPEs in general, with more females than males working in this area. The sample analysed included HPEs from different health disciplines. However, there was no differentiation between academic staff and clinical educators.

This is also a sample with a high representation of people with disability; even though there are no data of the prevalence of disability in HPEs, it is likely people with lived experience may be more inclined to complete the survey. Because several channels were used to promote the survey, it is unknown how many HPEs received the invitation and we are unable to determine the response rate. Finally, without a standard definition, it is unclear who self‐identifies as a HPE. HPEs are involved in the education of health profession students across multiple settings, from university classrooms, lecture theatres, online environments to simulated scenarios and various WIL in clinical settings. HPEs can either have dominant educational identities or dominant practitioner identities; this may affect their preparedness and concerns. They may also have different workload allocations and training backgrounds. Nevertheless, despite these limitations, this survey forms one body of evidence that can be supplemented by other methodologies such as interviews and focus groups. It is important to highlight that 100 responses allowed the validation of the SACIE‐R (labelled as Concerns) scale.

## 6 Conclusion

Our study highlights both optimism and critical areas for development in disability inclusion within health professions education. Although HPEs generally demonstrate positive attitudes, legal awareness and a willingness to support students with disability, they also report a lack of training and confidence in doing so. Experience appears to build competence, but this leaves early‐career educators at a disadvantage, underlining the need for structured and supported professional development. Broader cultural attitudes, particularly in clinical education settings, continue to challenge inclusion, especially given the prevailing ‘capability imperative’ that equates competence with able‐bodiedness. Addressing these challenges requires systemic shifts in culture and practice: a challenge that may be addressed by the widespread implementation of targeted, mandatory training to support inclusive educational practices.

## Author Contributions

All authors were involved in the conceptualisation of this work. The first draft of the paper was prepared by G.G. and C.H. C.H. performed the initial data analysis. All authors edited and reviewed the manuscript.

## Funding

This work was supported by a Research Grant from the Australian and New Zealand Association for Health Professional Educators.

## Ethics Statement

This study received approval by the Human Research ethics committees of the University of Notre Dame Australia (2023‐138S) and The University of Western Sydney (RH16139).

## Conflicts of Interest

The authors declare no conflicts of interest.

## Supporting information


**Data S1:** Supporting Information.

## Data Availability

The data that support the findings of this study are available on request from the corresponding author. The data are not publicly available due to privacy or ethical restrictions.

## Endnotes

^1^
We have chosen to use the terms ‘people with disability’ and ‘people with lived experience of disability’. However, we acknowledge that this language may not be consistent with preferences among disability stakeholders across the globe.
