# Supplementary material for: Health Professions' Educators' Preparedness for Inclusive Education: The HEPIE Study
Source: Clin Teach. 2026 Feb 17;23(2):e70364. doi: 10.1111/tct.70364 (PMC12911471; doi:10.1111/tct.70364)
Supplement: Supplementary file 1 — Data S1: Supporting Information. [file TCT-23-e70364-s001.doc]

## Supplementary Material. Adaptation of the Sentiments, Attitudes and Concerns about Inclusive Education Scale Revised (SACIE-R) to Health professions education

|  | **Original statement** | **Adapted statement** |
| --- | --- | --- |
| **1** | I am concerned that students with disabilities will not be accepted by the rest of the class. | I am concerned that learners with disability will not be accepted by other healthcare/clinical educators. |
| **2** | I dread the thought that I could eventually end up with a disability. | Not included deemed not appropriate |
| **3** | Students who have difficulty expressing their thoughts verbally should be in regular classes. | Not included not applicate for health professions education |
| **4** | I am concerned that it will be difficult to give appropriate attention to all students in an inclusive classroom. | I am concerned that it will be difficult to give appropriate support to learners with disability |
| **5** | I tend to make contacts with people with disabilities brief and I finish them as quickly as possible. | Not included deemed not appropriate |
| **6** | Students who are inattentive should be in regular classes. | Not included not applicate for health professions education |
| **7** | I am concerned that my workload will increase if I have students with disabilities in my class. | I am concerned that my workload will increase if I have learners with disability in my class, tutorial or clinical placement. |
| **8** | Students who require communicative technologies (e.g. Braille / sign language) should be in regular classes. | Learners who require technologies (e.g. screen readers, speech recognition software) should be able to participate in learning activities. |
| **9** | I would feel terrible if I had a disability. | Not included deemed not appropriate |
| **10** | I am concerned that I will be more stressed if I have students with disabilities in my class. | I am concerned that I will be more stressed if I have learners with disability in my class, tutorial or clinical placement. |
| **11** | I am afraid to look directly at a person with a disability. | Not included deemed not appropriate |
| **12** | Students who frequently fail exams should be in regular classes. | Not included not applicate for health professions education |
| **13** | I find it difficult to overcome my initial shock when meeting people with severe physical disabilities. | Not included |
| **14** | I am concerned that I do not have the knowledge and skills required to teach students with disabilities. | I am concerned that I do not have the knowledge and skills required to teach learners with disability |
| **15** | Students who need an individualized academic program should be in regular classes. | Not included not applicate for health professions education |
